# Fabrication of an Electrochemical Sensor Based on a Molecularly Imprinted Polymer for the Highly Sensitive and Selective Determination of the Antiretroviral Drug Zidovudine in Biological Samples

**DOI:** 10.3390/mi14101881

**Published:** 2023-09-30

**Authors:** Leyla Karadurmus, Sibel A. Ozkan

**Affiliations:** 1 Faculty of Pharmacy, Department of Analytical Chemistry, Adıyaman University, Adıyaman 02040, Türkiye; 2 Faculty of Pharmacy, Department of Analytical Chemistry, Ankara University, Ankara 06560, Türkiye; ozkan@pharmacy.ankara.edu.tr

**Keywords:** molecularly imprinted polymers, Zidovudine, voltammetry, photopolymerization

## Abstract

Molecularly Imprinted Polymers (MIP) have demonstrated considerable potential when combined with electrochemical sensors, exhibiting high sensitivity, selectivity and reproducibility levels. The aim of this work is to detect Zivudine (ZDV) in serum samples by means of an interface imprinting technique-based electrochemical sensor. Thus, ZDV was used as a template for the creation of an MIP-based electrochemical sensor, and differential pulse voltammetry (DPV) was used as the determination technique for the molecule. Electrochemical impedance spectroscopy (EIS) and cyclic voltammetry (CV) techniques were also used to characterize the electrochemical sensor capabilities, which showed a good linearity between 1.0 × 10^−10^ M and 1.0 × 10^−9^ M. ZDV was detected with a detection limit of 1.63 × 10^−11^ M, while the recovery analysis of spiked serum samples demonstrated that the sensor was highly selective.

## 1. Introduction

Human immunodeficiency virus (HIV) is nowadays one of the most serious global health concerns, and has contributed to close to 40 million fatalities. However, with effective HIV prevention, treatment, diagnosis and expanded access to healthcare, this chronic disease has become manageable (even including opportunistic infections), allowing patients to live as long as a non-infected person. Antiretroviral medication combinations are among the treatment plans to manage HIV [1]. Zidovudine (ZDV) is a 3′-azido-substituted pyrimidine 2′,3′-dideoxyribonucleoside molecule with thymine as the nucleobase, being both an azide and a pyrimidine 2′,3′-dideoxyribonucleoside. It acts as an HIV-1 reverse transcriptase inhibitor, an antimetabolite and an antiviral drug. The U.S. Food and Drug Administration (FDA) has approved the prescription of ZDV for the treatment of adult, pediatric and newborn HIV infections, but is always administered together with other HIV medications [2,3,4].

ZDV analysis by electrochemical [5,6,7], spectroscopic [8,9,10] and chromatographic [11,12,13] techniques has been reported (Table 1). The methodologies for the determination of ZDV in regard to sample handling, cost and sensitivity have been widely discussed. The methodology for the detection of ZDV in biological fluids needs to be improved. Akbari Hasanjani and Zarei studied the electrochemical response of ZDV upon a modified pencil graphite electrode created using deoxyribonucleic acid/Au-Pt bimetallic nanoparticles/graphene oxide-chitosan (DNA/Au-Pt BNPs/GO-chit/PGE), with ZDV measured by applying differential pulse voltammetry (DPV) [14]. The proposed sensor displayed a wide linear dynamic range from 0.01 pM to 10.0 nM in the optimum conditions, with a detection limit of 0.003 pM. An electrochemical method based on differential pulse voltammetry was presented for determining ZDV using hanging mercury drop electrodes, as reported by Barone et al. [15]. The linear dynamic range of standards in the buffer went between the detection limit of 4.1 nm to 206.5 μm. Trnková et al. studied ZDV by square wave voltammetry (SWV) using a hanging mercury drop electrode (HMDE), obtaining a detection limit of ZDV, in the absence and presence of ssDNA (10 μg/mL), of 1 and 250 nM, respectively [16]. Rafati and Afraz fabricated a ZDV sensor based on a nanocomposite of silver nanofilm (Ag-NF) and multiwalled carbon nanotubes (MWCNTs) immobilized on a glassy carbon electrode (GCE) using cyclic voltammetry (CV) and linear sweep voltammetry (LSV) techniques. The amperometric method, under optimal conditions, was used to determine ZDV ranging from 0.1 to 400 ppm (0.37 μM–1.5 mM), with a low detection limit of 0.04 ppm (0.15 μM) [17].

Ag-MWCNT_GCE: Ag nanofilm-multiwalled carbon nanotubes modified glassy carbon electrode, DE: Diamond Electrode, DPASV: differential pulse anodic stripping voltammetric methods, DPE: Diamond Paste Electrode, Dİ-hollow core-shells-PGE: Double layered one-by-one imprinted hollow core-shells pencil graphite electrode, DNA/Au-Pt BNPs/GO-chit/PGE: a modified pencil graphite electrode was made using deoxyribonucleic acid/Au-Pt bimetallic nanoparticles/graphene oxide-chitosan, HMDE: hanging mercury drop electrode, LSV: linear sweep voltammetry, MIP-core-shells-PGE: molecularly imprinted polymer-based core-shells (solid and hollow) @ pencil graphite electrode.

High sensitivity, low-cost, ease-of-use and fast sensing capabilities are some of the benefits that many researchers have identified when using electrochemical methods to measure medical substances. The key drivers behind the search for new electrochemical sensors’ development are the deficiencies associated with the current analytical methods, which lack in parameters such as sensitivity, selectivity and stability. As the sensors must respond to analytes in complicated matrixes, the selectivity of the sensors is one of the most crucial characteristics [21].

On the “Key-Lock” paradigm, MIPs have active sites where the substrate’s three-dimensional structure ends, allowing it to act just like a key. Polymers with specific target recognition sites are usually synthesized using molecular imprinting methods. Right after the polymerization, the target is washed out of the polymer to later create gaps on the polymer structure, where it can be recognized. In the right circumstances, these gaps allow the analyte molecule to bind there when its size, shape and chemical characteristics are recognized. In the subsequent steps, the MIP will detect the analyte molecule thanks to the generated active recognition sites, so the target will thereby be reattached to the polymer when it is present in a solution and this is mixed with the MIP [22,23,24]. Depending on how the functional monomer interacts with the template molecule, mainly two different methods—covalent or non-covalent—can be used to generate an MIP. When covalent bonding takes place between the functional monomer and the target, the bonds created during polymerization are the ones ruptured and eliminated from the polymer to create the MIPs. On the other hand, functional monomers that can interact non-covalently with the target, such as hydrogen bonds and van der Waals interactions, create communication between the functional monomer and the template molecule. A solvent is used to extract the template molecule from the polymer when the polymerization is complete [25,26,27,28].

In this work, a new MIP to be applied on an electrochemical sensor for the detection of ZDV was developed. We used acrylamide (ACR), hydroxyethyl methacrylate (HEMA) and crosslinker ethylene glycol dimethacrylate (EGDMA) for MIP synthesis. The MIP-based electrochemical sensor was fabricated directly on a GCE surface using the photopolymerization (PP) method, and 2-hydroxy-2-methylpropiophenone was used as a photoinitiator. Due to the high-selectivity capabilities of MIPs, the sensor for ZDV determination was effectively carried out, exhibiting good levels of selectivity and sensitivity. Two electrochemical approaches were used to describe the developed sensor, electrochemical impedance spectroscopy (EIS) and cyclic voltammetry (CV). It must be highlighted that the analysis procedure can be quickly completed without sophisticated or expensive equipment. Moreover, the proposed ACR-ZDV-MIP/GCE sensor has been successfully used to detect ZDV in complex matrixes: commercial human serum samples.

## 2. Experimental

### 2.1. Reagents and Chemicals

Glaxo Smith Kline provided ZDV’s main ingredients. The substances used in the work were of analytical purity and without further processing. The 1 × 10^−2^ M stock solution of ZDV was prepared in Methanol. Potassium ferricyanide (K_4_[Fe(CN)_6_], ≥99.0%) and potassium ferrocyanide (K_3_[Fe(CN)_6_], ≥98.5%) were provided by Merck. Methanol (99.8%), ethylene glycol dimethacrylate (EGDMA), Acrylamide (ACR), hydroxyethyl M-methacrylate (HEMA; ≥99%; >98.0%), 2-hydroxy-2 methyl propisphenone, 3-(trimethoxysilyl) propyl methacrylate (TMSPMA; ≥97.0%), sodium hydroxide (>97%), sodium acetate trihydrate (>99%), acetic acid (99%) (HAc), sodium phosphate monobasic (≥99.0%), paracetamol, ascorbic acid, potassium nitrate (KNO_3_), sodium sulfate (Na_2_SO_4_), magnesium chloride (MgCI_2_), dopamine and sodium dihydrogen phosphate dihydrate were purchased from Sigma-Aldrich. They were kept in the refrigerator to preserve the freshness of all the chemicals and stock solutions used in the experiments.

### 2.2. Equipment

The electrochemical experiments of the CV, DPV and EIS methods were carried out using AUTOLAB NOVA 2.1.4 software. Electrochemical measurements were performed in a three-electrode electrochemical cell. Pt wire was used as a counter electrode, a saturated Ag/AgCl (3 M KCl) was used as a reference electrode and GCE was used as a working electrode. For the cleaning, the GCE surface was physically (polishing kit) cleaned before each measurement, and 5 mM [Fe(CN)_6_]^3-/4-^ was used as the measuring solution.

The materials used in the experiments were weighed on a precision balance (Ohaus Instruments, Shanghai, China). pH measurements of the prepared solutions were made with a combined glass electrode pH meter (Mettler-Toledo pH/ion S220, Sonnenbergstrasse 74, CH-8603 Schwerzenbach, Switzerland). A Thermo-Shaker (Biosan TS-100) was used in the removal (RM) and rebinding (RB) stages of the sensor. All experimental studies were performed at room temperature (25 °C).

### 2.3. Fabrication of the ACR-ZDV-MIP/GCE Sensor

Before starting each experiment, the GCE was sonicated in (1:1) ethanol and ultrapure water for 10 min. Then, GCE was polished by dripping alumina on the polishing pad and subsequently rinsing it with ultrapure water. It dried at room temperature.

**Figure 1:**
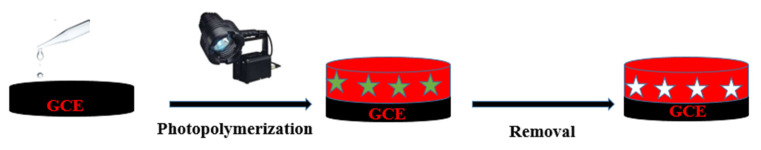
Schematic illustration of the ACR-ZDV-MIP/GCE sensor fabrication.

The proposed ACR-ZDV-MIP/GCE sensor was developed on GCE by copolymerizing ACR and HEMA with crosslinking EGDMA in the existence of ZDV. Under optimum conditions, the developed ACR-ZDV-MIP/GCE sensor was fabricated by keeping it under a UV lamp at room temperature (Figure 1). After confirming the presence of the film formed on the electrode surface, a Thermo Shaker was used for 5 min to remove the template molecule ZDV.

Experimental parameters such as the optimal template:monomer ratio, removal solutions, dropping volume, polymerization time, removal and rebinding time were determined for our new MIP-based sensor. The imprinting factors (IFs) were calculated using substances with similar chemical structures to analyze the sensor’s selectivity. The experimental outcomes indicated that the sensor proposed for ZDV was selective and sensitive in commercial human serum samples. The control measurements were applied using a non-imprinted polymer (NIP) prepared without adding ZDV with the same process performed for ACR-ZDV-MIP/GCE.

### 2.4. Preparation of Synthetic Human Serum Samples

Commercial human serum was held frozen at −20 °C in a fridge until examination. A standard process was applied to prepare a stock serum solution. To prepare a stock serum sample, 3.6 mL of synthetic human serum, 5.4 mL of acetonitrile and 1 mL of ZDV were added to a centrifuge tube. Firstly, it was centrifuged at 4000 rpm for 20 min, and later, the supernatant was taken. At this point, serum proteins were precipitated using acetonitrile. The supernatant was diluted with a supporting electrolyte in order to prepare specific amounts of the supernatant for the recovery tests. Each measurement was used at least three times for calibration experiments and for recovery experiments five times. To prove the accuracy of the sensor, recovery studies were carried out in serum and tablets. Recovery results were obtained by adding pure ZDV solution to the commercial serum at a known concentration. In addition, these results were obtained using DPV.

## 3. Results and Discussion

### 3.1. Electrochemical Characterization of the ACR-ZDV-MIP/GCE Sensor

**Figure 2:**
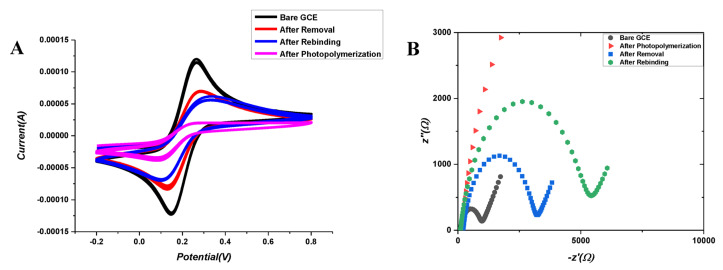
(**A**) CV and (**B**) EIS results of bare GCE after PP, after removal treatment and after rebinding of ZDV in 5 mM [Fe(CN)_6_]^3−/4−^ solution (0.1 M KCl).

For the electrochemical characterization of the developed ACR-ZDV-MIP/GCE sensor, EIS and CV methods were applied compared to the results of bare GCE after the photopolymerization, removal and rebinding of ZDV. These methods are basic techniques that describe the conductivity of the electrode surface, the change transfer resistance and the electron transfer process. The electrochemical behavior of the ACR-ZDV-MIP/GCE was carried out using a 5 mM [Fe(CN)_6_]^3−/4–^ solution as the redox probe. The sensors were characterized by evaluating the peak current and resistance values obtained during the preparation stages, such as bare GCE, after polymerization, after removal and after rebinding. The EIS and CV plots for each step of the PP processes are given in Figure 2. The current values for bare GCE and ACR-ZDV-MIP/GCE were 140 µA and 0.3 µA, respectively, using a five mM [Fe(CN)_6_]^3−/4−^ solution by DPV. In Figure 2A, the peak current values of the [Fe(CN)_6_]^3−/4−^ peak current were obtained as the maximum as there are no factors preventing electron transfer on the bare GCE surface (black line). After the polymerization processes, the peak intensity of the [Fe(CN)_6_]^3−/4−^ peak current was lost as the GCE surface was covered with a polymeric film, preventing electron transfer (pink line). After the removal of the ZDV molecule from the polymeric film, specific cavities to ZDV were formed, and the [Fe(CN)_6_]^3−/4−^ peak current observed was lower than that of bare GCE (red line). Finally, the peaks obtained by rebinding the known concentration of ZDV were lower than those after the removal due to the closure of the voids on the polymeric film (blue line). In the EIS measurements, Nyquist plots and changes in charge transfer resistance (Rct) were evaluated (Figure 2B), with the same steps applied. According to the EIS results, the bare GCE surface had the lowest Rct values (black dots) since electron transfer can be easily realized there. Since the formation of polymeric films on the GCE surface after polymerization cancels the electron transfer, Rct values (red dots) reach the highest value. After ZDV molecules are removed, specific cavities are formed on MIP surfaces, and Rct values (blue dots) decrease when electron transfer takes place. However, with the rebinding of the ZDV, the gaps are partially filled, making it difficult for the electron transfer to happen, thus causing the Rct values (green dots) to rise again. It confirms that ZDV molecules selectively bind to specific gaps in the developed MIP sensors.

Furthermore, we used the Randles-Sevcik equation (Ip = 2.69 × 105 n^3/2^ AD^1/2^ υ½ C) to calculate the electroactive surface areas of GCE before photopolymerization, after photopolymerization, after removal and after rebinding. In this formula, Ip stands for the peak current, n stands for the number of transferred electrons (calculated as 1 for potassium ferri/ferrocyanide), A stands for the active surface area (cm^2^), D stands for the diffusion coefficient (calculated as 7.6 × 10^−6^ cm^2^s^−1^ for potassium ferri/ferrocyanide), υ stands for the scan rate and C stands for the concentration of the probe. As a result of the calculations, the electroactive surface area of GCE is 0.117 cm^2^ before polymerization, 0.00025 cm^2^ after polymerization, 0.075 cm^2^ after removal and 0.05 cm^2^ after rebinding. These outcomes can be represented by the coating of the surface after polymerization and the decrease in the active surface area.

### 3.2. Optimization of MIP Fabrication

Evaluating and optimizing the molecular imprinted film is the most crucial part of MIP-based sensor fabrication. The results of some optimization parameters affecting the sensor response are given in Table 2.

#### 3.2.1. Template/Monomer Ratio

**Figure 3:**
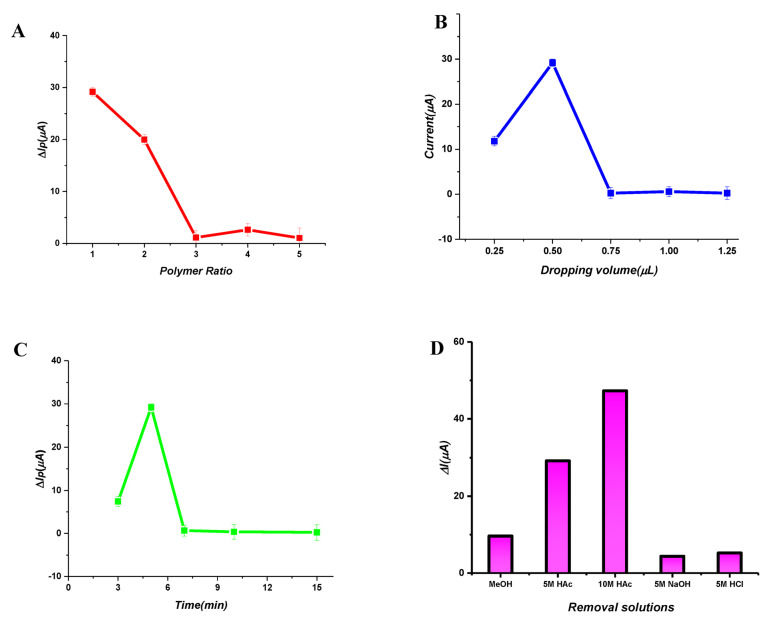

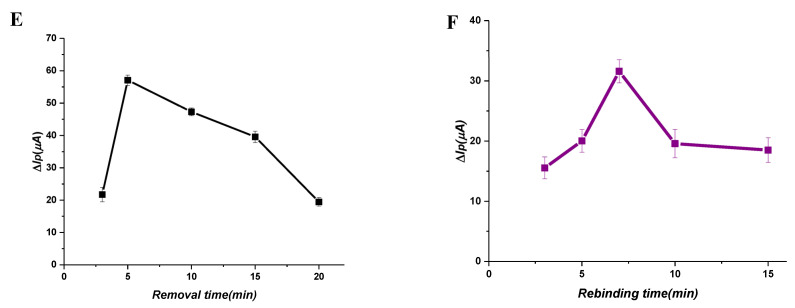
Optimization of monomer/template ratio (**A**), dropping volume (**B**) and photopolymerization time (**C**). Effect of removal solutions (**D**) and removal times (**E**) with the plot of ΔIp values after removal and after rebinding of ZDV. The plot of ΔIp values after removal and after rebinding of ZDV versus different rebinding times (**F**).

Optimizing the template:monomer ratio is critical to fabricating efficient and stable polymeric films. Since the creation of polymeric films is directly connected to the interactions between the template and the monomer, the optimal ratio should be determined. Also, the template:monomer ratio can enhance sensor selectivity in molecular imprinting techniques. However, a non-selective electrochemical reaction for the template can result from using an excess of the monomer, which could also deform the imprinting sites. Therefore, different monomer:template ratios (1:1, 2:1, 3:1, 4:1 and 5:1) were evaluated for the PP preparation of polymeric films. The differences between the peak currents obtained after removal and polymerization were calculated (Figure 3A). According to the ΔI values, the 1:1 ratio was chosen as the optimum value for both sensors, which provides the most stable and efficient polymer.

#### 3.2.2. Dropping Volume

The dropped volume onto the GCE surface is directly related to the thickness of the polymeric film and the polymer formation procedure; hence, it must be optimized. Thus, different dropping volumes (0.25–1.25 µL) were dropped onto the GCE surface, so that the developed ACR-ZDV-MIP/GCE sensor could be optimized in order to obtain the best polymeric film in terms of conductivity, diffusion limitation and thickness. It was evaluated by taking the difference between the peak currents obtained after removal and after polymerization, and the optimum dropping volume was obtained as 0.50 µL (Figure 3B).

#### 3.2.3. Polymerization Time

The ACR-ZDV-MIP/GCE sensor was obtained using a UV lamp with a wavelength and power density of 365 nm and 100 W, respectively. Optimizing the PP time under a UV lamp forms a stable polymeric film on the GCE surface. After 0.50 µL of polymerization solution was dropped onto the GCE surface, it was exposed to UV light for 3–15 min. Then, the differences in the peak currents after removal and after PP were compared. Considering that the sensor preparation time must be ideally short, 5 min of UV light exposure was chosen as the optimum polymerization time (Figure 3C).

#### 3.2.4. Removal Solution and Time

Removing template molecules from MIP films without damaging the polymeric film is a critical step. In this part, specific cavities are formed after the template molecule is removed, and the analyte is easily attached to these cavities. Therefore, suitable removal solutions were selected. For PP, different removal solutions, such as acetone, MeOH, HAc (10.0 M), NaOH (5.0 M) and HCl (1.0 M), were tested. It was evaluated by taking the difference between the peak currents of the solvents after removal and after PP. When the effects on the removal process were examined, the peak currents obtained using HAc (10.0 M) were higher than those of other solvents and were chosen as the removal solvent (Figure 3D). In the removal process, the modified sensor immersed in 10.0 M HAc was incubated at different times (3–20 min) using a ThermoShaker (600 rpm, 25 °C). The removal time was evaluated by taking the difference between the peak currents after removal and after PP. The best removal time for PP was chosen as 5 min (Figure 3E).

#### 3.2.5. Rebinding Time

The rebinding procedure is a significant parameter for determining the analysis time and performance. Therefore, the MIP-based sensor prepared by PP was immersed in an 8 × 10^−11^ M ZDV solution to evaluate its effect on reattachment at different times (3–15 min), and was examined using a ThermoShaker (600 rpm, 25 °C). When the difference between the after-rebinding and after-removal peak currents was evaluated, it was seen that ΔI remained almost the same after reaching its highest value after 7 min. Therefore, according to Figure 3F, the rebinding time was selected as 7 min for the ACR-ZDV-MIP/GCE sensor. 

### 3.3. Analytical Validation of ACR-ZDV-MIP/GCE Sensor

**Figure 4:**
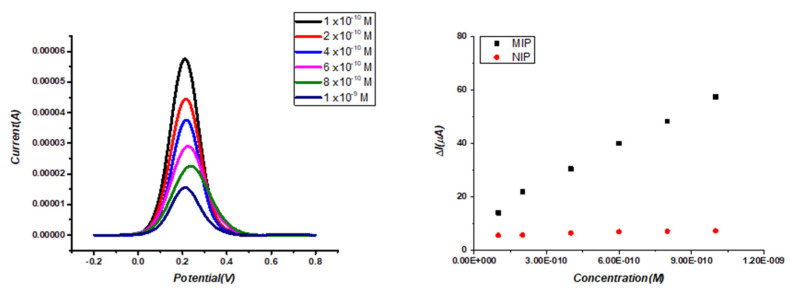
DPV of ZDV on ACR-ZDV-MIP/GCE (**A**) A linear relationship between the ΔI and ZDV concentrations in ACR-ZDV-MIP/GCE and NIP/GCE (**B**).

The analytical performances of the ACR-ZDV-MIP/GCE sensor were evaluated using different concentrations of ZDV in a standard solution under optimum conditions. The linearity range was between 1.0 × 10^−10^ and 1.0 × 10^−9^ M using the DPV technique in 5.0 mM [Fe(CN)_6_]^3-/4-^ solution. DP voltammograms and linear curves of this sensor were given in Figure 4A. A linear curve was obtained using ΔI values versus different ZDV concentrations. The calibration equation of the ACR-ZDV-MIP/GCE sensor was found as ΔI = 5.0 × 10^10^ × C + 11.247 (r = 0.995) (Table 3). C is the concentration of ZDV. LOD and LOQ values were calculated using the formulas 3× s/m and 10× s/m (s: standard deviation, m: slope) and were determined as 1.63 × 10^−11^ and 5.42 × 10^−11^ M, respectively.

The analytical performances of the ACR-ZDV-MIP/GCE sensor were tested with NIP sensors prepared in the absence of ZDV in Figure 4B. The results showed that the developed sensors have excellent sensitivity to detect ZDV.

### 3.4. Application of ACR-ZDV-MIP/GCE Sensor in Commercial Human Serum

To assess its feasibility and potential, the MIP-based electrochemical sensor was applied to detect ZDV in biological samples. Table 2 displays the calculated accuracy and precision values. These results indicate that the developed sensor is suitable for analyzing ZDV in serum samples. The responses of the ACR-ZDV-MIP/GCE sensor were linear in different ZDV concentration ranges in commercial human serum samples (Table 3), and the linear regression equations of the developed sensors are given below:

ΔI = 6.0 × 10^10^ × C + 20.588 (r = 0.996)

**Figure 5:**
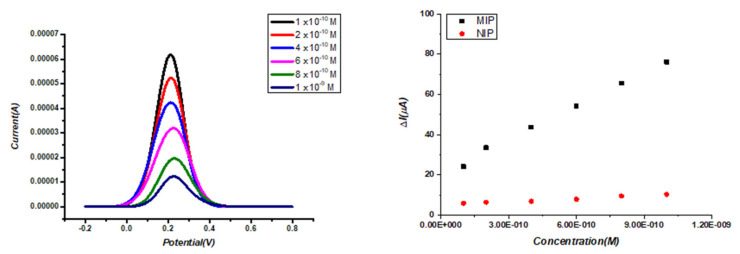
In spiked serum samples, DPV of ZDV on ACR-ZDV-MIP/GCE (**A**) A linear relationship between the ΔI and ZDV concentrations in ACR-ZDV-MIP/GCE and NIP/GCE (**B**).

DPV voltammograms obtained using ACR-ZDV-MIP/GCE as standards are shown in Figure 5A. The NIP-based sensors were also used to validate the applicability of the MIP-based sensors, and the nonlinearity of the NIP-based sensors versus the MIP-based sensors was observed (Figure 5B). Excellent recovery and %RSD results in commercial human serum samples, also with satisfactory accuracy and applicability, were obtained with the ACR-ZDV-MIP/GCE sensor (Table 4).

### 3.5. The Selectivity Studies

**Figure 6:**
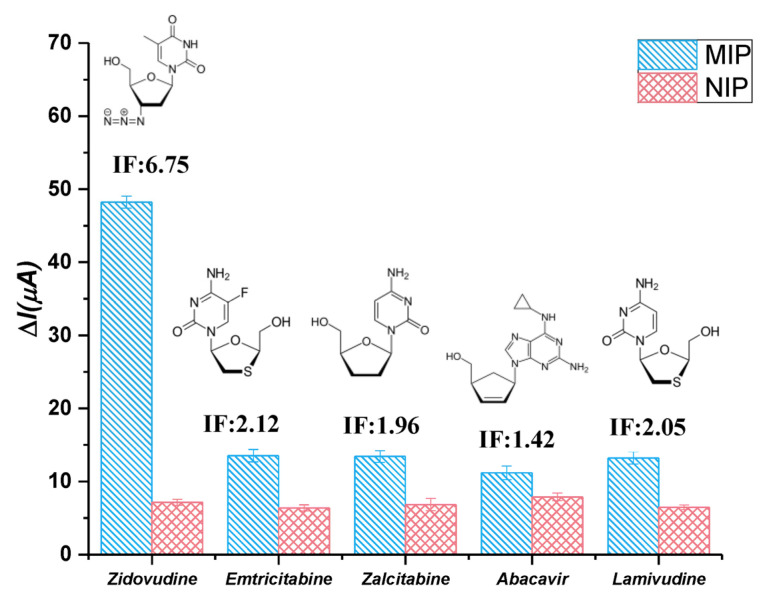
Molecular structures and selectivity values of ZDV, Emtricitabine, Zalcitabine, Abacavir and Lamivudine on ACR-ZDV-MIP/GCE.

One of the characteristics of an ideal sensor is its selectivity toward other species. To evaluate the selectivity of the developed sensor, the electrochemical responses of MIP and NIP electrodes for ZDV and other substances were examined using the DPV technique, and the obtained results are presented in Figure 6. The selectivity of the interfering species was measured intuitively using the imprinting factor (IF) = ΔI(MIP)/ΔI(NIP). ZDV exhibited an IF value of 6.75, as shown in Figure 6, while the other interfering species had IF values between 2.12, 1.96, 1.42 and 2.05 for Emtricitabine, Zalcitabine, Abacavir and Lamivudine, respectively. The results indicate that the proposed electrochemical sensor is more selective and sensitive to ZDV molecules than to other interfering species. The ability of the MIP layers to selectively identify target analytes was excellent, and the selectivity of the proposed sensor was found to be sufficient.

### 3.6. Interference Studies

Response interferences due to other molecules and ions during the detection of ZDV were assessed. Besides studying the selectivity against structurally similar compounds, the efficiency of MIP-based sensors may also be influenced by some other compounds in biological fluids, such as K^+^, Cl^–^, Na^+^, SO_4_^2–^, dopamine (DA), ascorbic acid (AA), uric acid (UA) and paracetamol (PAR). For the selectivity test, the ZDV concentration in the MIP/GCE was used as 2.5 × 10^−11^ M. The RSD and recovery values were determined in the presence of 10 times more interference agents. The RSD values for the MIP/GCE were found as ≤1.85% (Table 5). These results presented that in the presence of interference, agents did not significantly affect the performance of the sensors developed for ZDV detection.

### 3.7. Stability

**Figure 7:**
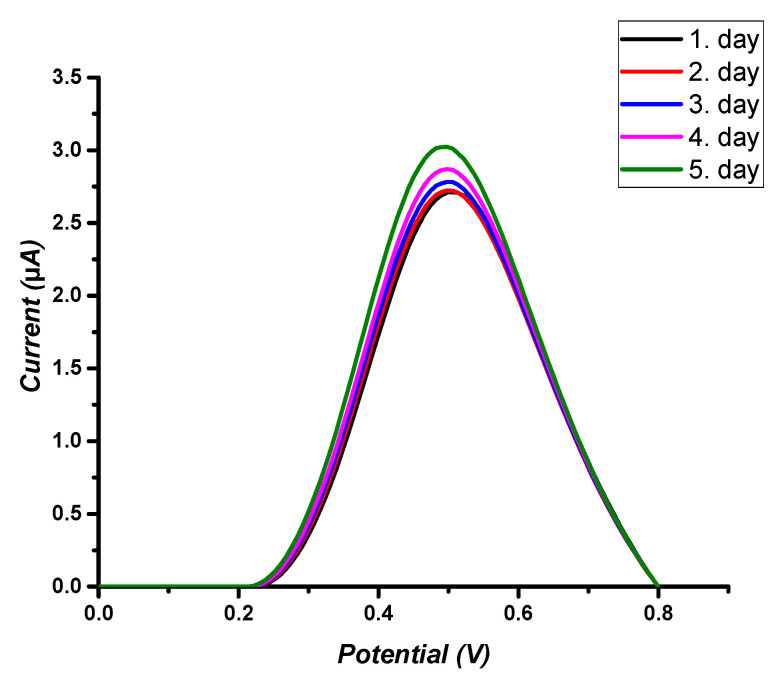
Storage stability of the ACR-ZDV-MIP/GCE sensors.

Storage stability experiments indicated the applicability and reusability of the ACR-ZDV-MIP/GCE sensors. As a result of these experiments, it has been discovered that effective results can be obtained in up to 5 days (Figure 7). 

## 4. Conclusions

Here, we present a novel electrochemical sensor based on MIP to detect ZDV in commercial human serum samples. The suggested ACR-ZDV-MIP/GCE sensor demonstrated a linear range between 1.0 × 10^−10^ M and 1.0 × 10^−9^ M, a low detection limit of 1.63 × 10^−11^ M and a selective discrimination of ZDV from structural analogs under ideal working circumstances. The proposed ACR-ZDV-MIP/GCE sensor also offers considerable advantages over traditional detection methods. It can be regenerated in situ and used for continuous analysis without being time-consuming. When it was used to quantify ZDV in serum samples, the newly constructed ACR-ZDV-MIP/GCE sensor showed good recoveries and a satisfactory accuracy.

## Figures and Tables

**Table 1:** Electroanalytical studies about ZDV.

Electrode	Method	Linear Range	Limit of Detection	Ref.
DNA/Au-Pt BNPs/GO-chit/PGE	DPV	0.01 pM to 10.0 nM	0.003 pM	[14]
HMDE	DPV	4.1 nM to 206.5 microM	4.1 nM	[15]
DPE	Amperometric	4 × 10^−4^ to 6 × 10^−2^ fmol/L	2 × 10^−4^ fmol/L	[18]
DE	-	1–100 ng/ml	1 ng/ml	[19]
HMDE	SWV	1 and 250 nM	10 µg/ml	[16]
HMDE	DPV	0.25 to 1.25 mg/L	0.0025 mg/L	[20]
MIP-core-shells-PGE	DPASV	4.76–128.76 ng mL^−1^	1.26 ng mL^−1^	[5]
Ag-MWCNT_GCE	LSV	0.37 μM–1.5 mM	0.15 μM	[17]
Dİ-hollow core-shells-PGE	DPASV	-	0.91 ng mL^−1^	[6]

**Table 2:** Results of some optimization parameters affecting the sensor response.

Parameters	Range	Selected Value
Template/Monomer ratio	1:1–1:5	1:1
Dropping volume(µL)	0.25–1.25	0.50
Polymerization time (min)	3–15	5
Removal time (min)	3–20	5
Rebinding time (min)	3–15	7

**Table 3:** Assay validation sheet of the ACR-ZDV-MIP/GCE sensors used to assay ZDV.

Parameter	Standard Solution	Serum Sample
Linearity range (M)	1 × 10^−10^–1 × 10^−9^	1 × 10^−10^–1 × 10^−9^
Slope (μA M^−1^)	5.0 × 10^10^	6.0 × 10^10^
Intercept (µA)	11.247	20.588
SE of intercept	2.124	2.431
Correlation coefficient (*r*)	0.995	0.996
LOD (M)	1.63 × 10^−11^	1.36 × 10^−11^
LOQ (M)	5.42 × 10^−11^	4.54 × 10^−11^
Repeatability of peak current (RSD%) ^a^	0.893	1.034
Reproducibility of peak current (RSD%) ^a^	1.376	1.521

^a^ Each value is an average of three measurements.

**Table 4:** Recovery studies for commercial serum samples.

	PP
Spiked amount (mg)	10.00
Found amount (mg) *	9.96
Average recovery (%)	99.6
RSD%	1.23
Bias%	0.40

* Each value is the mean of five experiments.

**Table 5:** Interference effect of different compounds on ZDV determination.

Interferences	Recovery of ZDV (%)	RSD (%)
KNO_3_	100.8	2.01
Na_2_SO_4_	99.6	2.06
MgCl_2_	99.8	0.64
DOP	99.9	1.73
AA	103.9	1.09
UA	98.6	1.71
PAR	100.8	2.04

## Data Availability

Not applicable.
